# External validity of randomized controlled trials in older adults, a systematic review

**DOI:** 10.1371/journal.pone.0174053

**Published:** 2017-03-27

**Authors:** Floor J. van Deudekom, Iris Postmus, Danielle J. van der Ham, Alexander B. Pothof, Karen Broekhuizen, Gerard J. Blauw, Simon P. Mooijaart

**Affiliations:** 1 Department of Gerontology and Geriatrics, Leiden University Medical Centre, Leiden, The Netherlands; 2 Institute for Evidence-Based Medicine in Old Age (IEMO), Leiden, The Netherlands; 3 Division of Vascular and Endovascular Surgery, Department of Surgery, Beth Israel Deaconess Medical Center, Boston, Massachusetts, United States of America; Universitat de Valencia, SPAIN

## Abstract

**Background:**

To critically assess the external validity of randomized controlled trials (RCTs) it is important to know what older adults have been enrolled in the trials. The aim of this systematic review is to study what proportion of trials specifically designed for older patients report on somatic status, physical and mental functioning, social environment and frailty in the patient characteristics.

**Methods:**

PubMed was searched for articles published in 2012 and only RCTs were included. Articles were further excluded if not conducted with humans or only secondary analyses were reported. A random sample of 10% was drawn. The current review analyzed this random sample and further selected trials when the reported mean age was ≥ 60 years. We extracted geriatric assessments from the population descriptives or the in- and exclusion criteria.

**Results:**

In total 1396 trials were analyzed and 300 trials included. The median of the reported mean age was 66 (IQR 63–70) and the median percentage of men in the trials was 60 (IQR 45–72). In 34% of the RCTs specifically designed for older patients somatic status, physical and mental functioning, social environment or frailty were reported in the population descriptives or the in- and exclusion criteria. Physical and mental functioning was reported most frequently (22% and 14%). When selecting RCTs on a mean age of 70 or 80 years the report of geriatric assessments in the patient characteristics was 46% and 85% respectively but represent only 5% and 1% of the trials.

**Conclusion:**

Somatic status, physical and mental functioning, social environment and frailty are underreported even in RCTs specifically designed for older patients published in 2012. Therefore, it is unclear for clinicians to which older patients the results can be applied. We recommend systematic to transparently report these relevant characteristics of older participants included in RCTs.

## Introduction

Older individuals are often underrepresented in randomized clinical trials (RCTs).[1–3] They are frequently excluded as a result of direct and indirect exclusion criteria based on the presence of comorbidities and polypharmacy.[4] For instance, Van de Water et al. previously demonstrated that due to exclusion criteria based on age, comorbidities and medical history only a maximum of 12% of older breast cancer patients would have been suitable to enter breast cancer trials.[5] The consequence is that participants enrolled in clinical trials often do not represent the older patients in general medical practice and thus threaten the external validity of RCTs in the older patient population.[6, 7]

Compared to younger patients, older patients are very heterogenic with respect to frailty, mobility, functional capacity, and cognitive function. These different domains can be systematically assessed by using geriatric assessments.[8] To critically interpret the outcome in RCTs and to allow clinicians to judge to which older patients the outcomes can be applied, it is important to know which older adults have been enrolled in the trials. In scientific literature, patient characteristics are usually described in the population descriptives or in the in- and exclusion criteria section. It is currently unknown how patient characteristics with respect to physical, mental and social functioning or frailty are reported in RCTs specifically designed for older adults.

Therefore, the aim of this systematic review is to study what proportion of RCTs specifically designed for older adults report on somatic status, physical and mental functioning, social environment and frailty in the patient characteristics.

## Methods

### Study selection

For the present study we used the sample from the previously published systematic review by Broekhuizen et al. showing that only 7% of the RCTs published in 2012 were specifically designed for older adults.[3] The complete search strategy was published previously. In short, a systematic search was conducted to identify RCTs that were published in 2012 (n = 26,740), and after removing duplicates a random sample was drawn (n = 2375). Articles were further excluded when it was not written in English, had no RCT design, when the study included non-human subjects or reported secondary analyses. After applying the exclusion criteria and retrieved full-text, 1369 identified articles remained. For the current review we started with the sample of 1369, we defined "specifically designed for older patients" as a mean age of trial participants of 60 years or older and we included all randomised controlled trials of which the mean age was 60 years or older.

### Data extraction

Items extracted from each study included: publication data (author, year), patient characteristics (sample size, median age, percentage of males, disease categories and geriatric assessments). Disease category was classified according to the International Classification of Diseases (ICD-10) of the World Health Organization (WHO). Two researchers (FvD, IP) extracted the geriatric assessments and in case of disagreement, consensus was reached after discussion with a third co-author (SPM).

### Geriatric assessments

For all studies we extracted if geriatric assessments were reported in the patient characteristics, which are usually reported in the population descriptives or in the in- and exclusion criteria section. The geriatric assessments were classified into five geriatric domains: somatic status, physical functioning, mental functioning, social environment and frailty. Somatic status was defined as the presence of assessments of somatic co-morbid diseases and polypharmacy. Co-morbid diseases had to be assessed by quantitative instruments that measure cumulative disease burden or quantitatively by adding up the number of chronic and acute medical illnesses. Polypharmacy had to be assessed by validated tools. Physical functioning was defined as assessments of functional performance, mobility, and objectively measured physical capacity such as hand grip strength, gait speed or balance tests. Mental functioning was defined as assessment of any domain within cognition, dementia diagnosis, and mood or depression. Assessments were classified to the social environment domain when they depicted information about the social support system (living alone or with partner, marital status, family care giver), domestic services (home help and care) and the way of living (self-reliant or community dwelling, assisted living or nursing home). Assessments were classified within the frailty domain when they were used as frailty index or instrument (for instance, Fried Frailty Phenotype, Rockwood Frailty Index, Groningen Frailty Indicator), which assessed the frailty status.

### Statistical analysis

Measures of central tendency of continuous variables from the trials were recorded as mean with standard deviation (SD) or median with interquartile range (IQR). For dichotomous variables the number of subjects with the characteristic divided by the total number of subjects was recorded. We plotted the proportion of trials in which either geriatric assessment was reported in the population descriptives or in the in- and exclusion criteria. As a sensitivity analyses we used different cut-offs for the definition of "specifically designed for older patients" using a minimum mean age of 70 years or 80 years instead of 60 years in the main analysis. All analyses were performed with IBM SPSS Statistics version 23.0.

## Results

**Fig 1:**
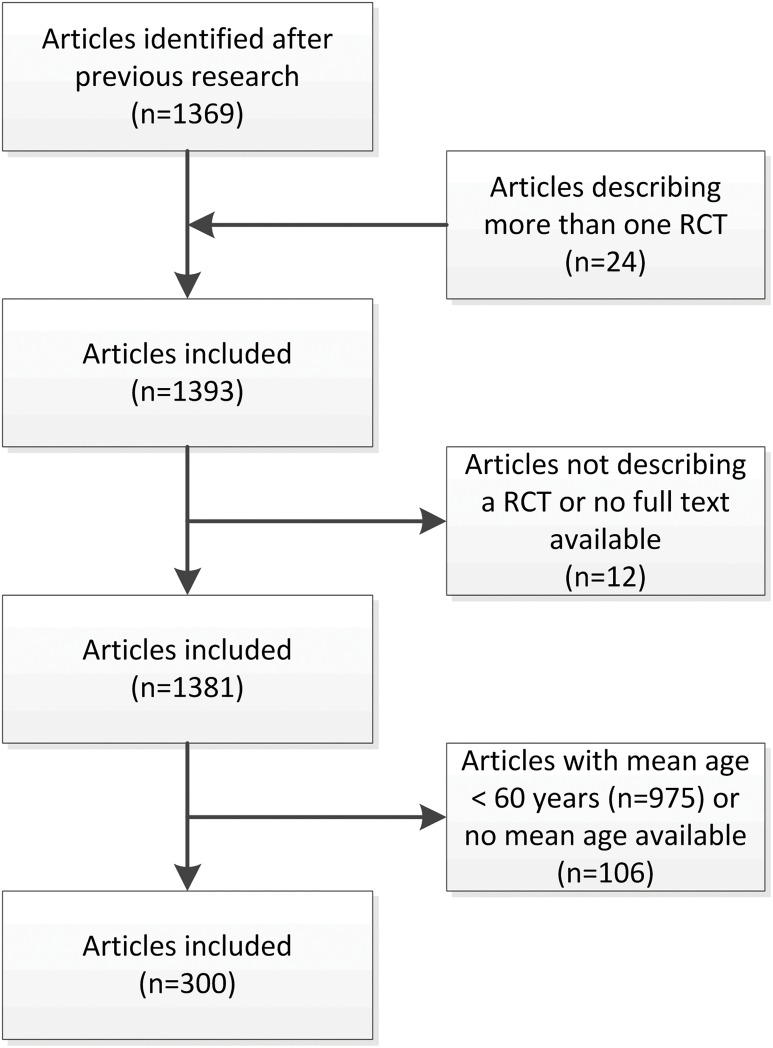
Flow chart for inclusion of studies. PRISMA flow chart of the result from the performed search strategy and selection process.

The analysis in the present review started with 1369 articles. Of these 1369 articles, some articles described more than one RCT (adding a total of 24 RCTs), articles were further excluded because there was no RCT design after second review (n = 11) or no full-text was available (n = 1). After all the articles with a mean age <60 years or the articles were no mean age was available were removed. We ended up with 300 articles specifically designed for older people included for this analysis. (Fig 1) A full database of all 300 included publications, including authors, titles and journal of publication can be assessed (S1 Appendix).

Table 1 shows a description of the main trial characteristics of these 300 trials. The median number of participants per trial was 114 (IQR 47–288), the median of the reported mean age of the participants in the trials was 66 (IQR 63–70) and the median percentage of men included in the trials is 60 (IQR 45–72). Most of the trials were classified into WHO disease categories circulatory (25%), neoplasms (19%), musculoskeletal (9%), nervous (8%) and digestive (6%).

**Table 1:** Main trial characteristics of the 300 included RCTs.

Main trial characteristics		n = 300
Number of participants, N (median, IQR^a^)		114 (47–288)
Age of participants, years (median, IQR)		66 (63–70)
Percentage men included in trial (median, IQR) ^b^		60 (45–72)
Disease categories, N (%)		
	Circulatory	74 (25)
	Neoplasms	56 (19)
	Musculoskeletal	28 (9)
	Nervous	23 (8)
	Digestive	19 (6)
	Other	100 (33)

^a^Interquartile range, difference between 25^th^ and 75^th^ percentile is reported

^b^Data are based on 288 (96%) trials

**Fig 2:**
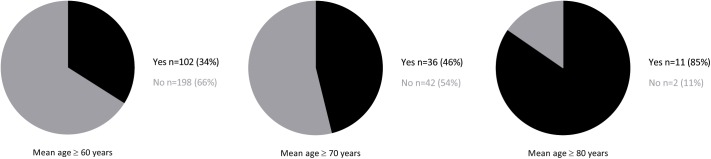
Proportion of RCT’s in older patients that report on geriatric assessments in the patient characteristics. Showing the proportion of trials reporting geriatric assessments in the population descriptives or in- and exclusion criteria.

**Fig 3:**
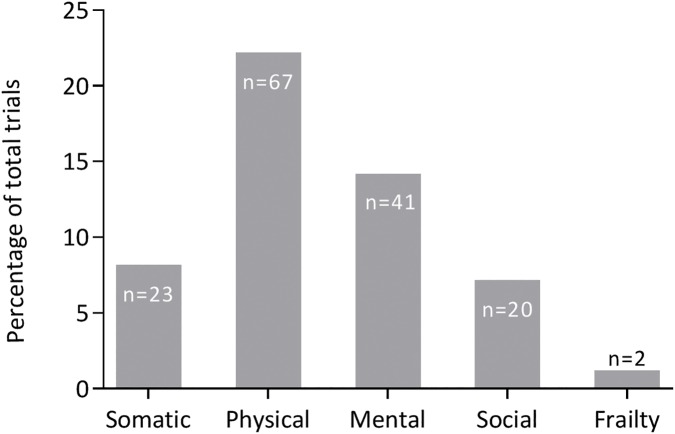
Proportion of RCT’s in older patients that report on different geriatric assessments*. Showing the distribution of different geriatric measurements and expressed as percentage of the total trials (n = 300). *Some articles reporting more than one domain: 14 articles reporting two geriatric domains, eight articles reporting three geriatric domains and only one article reports four geriatric domains.

Fig 2 shows the proportion of RCTs that reported on geriatric assessments in the patient characteristics. In 102 trials (34%) somatic status, physical and mental functioning, social environment or frailty were reported in the patient characteristics. In 73 trials (24%) these geriatric domains were reported in the in-, or exclusion criteria, and in 83 trials (28%) geriatric domains were reported in the population descriptives. In total of the 300 trials somatic status was reported 23 times (8%), physical functioning 67 times (22%), mental functioning 41 times (14%), social environment 20 times (7%) and frailty was only reported 2 times (1%). (Fig 3)

When selecting trials with a reported mean age of 70 years and older (n = 78), 46% of the trials report geriatric assessments in the patient characteristics. When selecting trials with a reported mean age of 80 years and older (n = 13), 85% of all trials report on geriatric assessments in the patients characteristics (Fig 2).

## Discussion

The main finding of this article is that only in 34% of all trials specifically designed for older patients report of geriatric assessment in the patient characteristics.

Our results are in line with the limited evidence that geriatric characteristics are underreported in RCTs. Benraad et al. described that geriatric characteristics are rarely taken into account in RCTs on anti-depressant drugs in late-life depression.[9] There are a number of possible explanations of the limited report of somatic, physical and mental functioning, social environment and frailty in RCTs published in 2012. First, the underreporting of somatic, physical and mental functioning, social environment and frailty might suggest that they were not taken into account at all. Second, it is possible that assessments of somatic, physical and mental functioning, social environment and frailty were included in the study protocol but were not reported in the published paper. This is also known from literature, describing that in 12% of the trials published in high-impact general medical journals the exclusion criteria were not well reported.[6] Third, the included participants in RCTs might have been implicitly selected based on protocol level, patient level or physician level. An example of protocol level is that the study protocol prescribes to visit the research facility three times a week. Older patients who have an impaired mobility or do not have a caregiver available, will be less likely to participate and are implicit selected on the functional or social domain. A form of implicit selection on patient level is a form of healthy user bias in which only the healthy older adults are willing to participate. Implicit selection on physician level is a phenomenon also described in literature, in which eighteen percent of the treating physicians stated that they had not offered their older patients a clinical trial because of comorbid conditions that might have affected their response to treatment, even though they had met the eligibility criteria for the trial.[10] In conclusion, as a result of the very limited report of somatic, physical and mental functioning, social environment and frailty, the external validity of the trial results is very limited. This might hamper the extrapolation of the trial results to individual older patients who suffer from functional impairment or frailty.

Literature describes that assessment of external validity is complex[11] but at least the characteristics of the included study population should be described in a transparent fashion[12] and therefore at least include patient and disease characteristics[13]. The included study population can be assessed by the description of the in- and exclusion criteria and patient and disease characteristics are usually found in the population descriptives. Especially in case of older adults, because of their huge heterogeneity as described previously, it is important to have a complete insight of the patient characteristics. We realise that insufficient time or funding can be one of the reasons not taking the geriatric assessment into account. However, this step has to be taken to gain better insight whether the results are applicable to older adults seen in regular practice[14, 15]. The choice of the domain assessed and instruments used depends on the patient population, the intervention and the outcome, unfortunately literature has no consensus on this point yet. From the present review we can conclude that it is currently difficult for the clinician to judge for which older adult the results of RCTs can be applied. This adds to the lack of evidence that already exists because of the very limited number of trials that specifically targets older patients.

We included only RCT's with a median age of 60 years or older. It is not expected that trials including younger adults perform geriatric assessments. Although the age of 60 years and older is chosen rather arbitrarily, it is striking that even in this sub-selection only one third of the trials reports on geriatric assessments to describe its population. Even when selecting the RCTs with a median age of 70 and older, not even half of the trials reporting on geriatric assessments. Only when selecting RCTs with a median age of 80 and older, the report on geriatric assessments 85%, however this is just representing less than one percent of all the included trials.

There are a few limitations to this systematic review. Our search was limited to a 10% random sample of the identified publications from 2012. However, since it contains a random sample, we can assume this is a representative sample, although we did not formally test this. Second, we excluded 106 articles in were no mean age was reported. The main strength of this review is that it is was currently not known how somatic status, physical and mental functioning, social environment and frailty are used and reported in RCTs. This review gains more insight in the external validity of RCTs for older adults.

## Conclusion

Somatic status, physical and mental functioning, social environment and frailty are underreported even in RCTs specifically designed for older patients published in 2012. Therefore, it is unclear for clinicians to which older patients the results can be applied. We recommend systematic to transparently report these relevant characteristics of older participants included in RCTs.

## Supporting information

S1 PRISMA Checklist (DOC)

S1 Appendix The 300 included publications, including authors, titles and journal of publication. (XLSX)
